# Biomarkers for immune checkpoint inhibitors in colorectal cancer: recent advances and future perspectives

**DOI:** 10.20892/j.issn.2095-3941.2023.0201

**Published:** 2023-09-14

**Authors:** Changjiang Yang, Long Zhao, Yilin Lin, Shan Wang, Yingjiang Ye, Zhanlong Shen

**Affiliations:** Department of Gastroenterological Surgery, Laboratory of Surgical Oncology, Key Laboratory of Colorectal Cancer Diagnosis and Treatment Research, Peking University People’s Hospital, Beijing 100044, China

Colorectal cancer (CRC) has become a major threat to human health. Recent years, improvements have been seen in the treatment of advanced CRC with immune checkpoint inhibitors (ICIs). Nonetheless, sensitivity to ICIs notably varies among patients, thus greatly limiting clinical applications of ICIs in CRC. Hence, the identification of biomarkers that can accurately distinguish between ICI-sensitive and drug-resistant patients is of utmost importance. Such biomarkers are essential for selecting appropriate treatment regimens and achieving precision therapy (**Figure 1**). The biomarkers discussed below provide insights into the advancements made in this field (**Table 1**).

**Figure 1:**
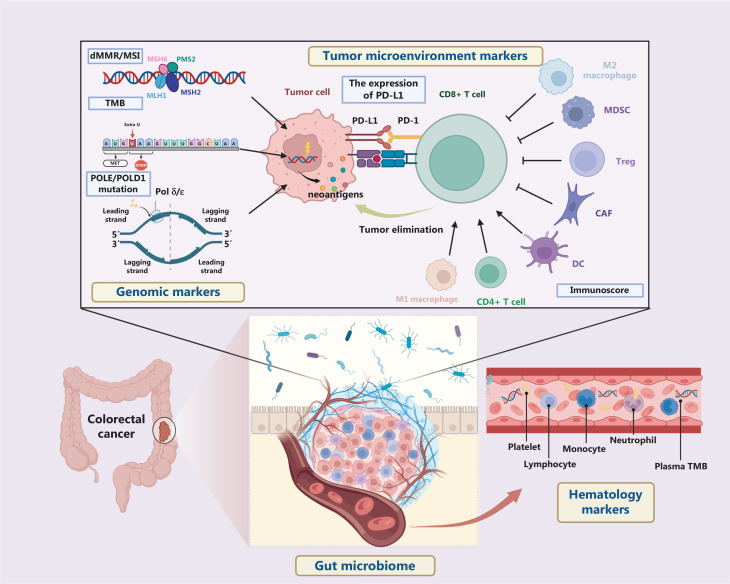
Predictive biomarkers for ICI treatment of colorectal cancer. The figure depicts the 4 aspects characterizing biomarkers that have been identified or might plausibly be used for ICI therapy in CRC: genomic markers, tumor microenvironment markers, hematology markers, and the gut microbiome.

**Table 1:** Summary of the main findings regarding biological biomarkers for ICI treatment of CRC

Category	Biomarkers	Samples	Evaluation methods	Highlights
Genomic markers	dMMR/MSI	Fresh or formalin-fixed, paraffin-embedded tumor samples	Immunohistochemistry, polymerase chain reaction (PCR), or next-generation sequencing	The primary foundation for assessing the effectiveness of ICIs in CRC
	TMB	Formalin-fixed paraffin-embedded tumor samples	Whole-exome sequencing, panel sequencing	Need for a consensus regarding the optimal standardization of TMB values across various platforms, and the threshold value for effectively identifying patients with high and low TMB
	POLE/POLD1 mutation	Formalin-fixed, paraffin-embedded tumors or fresh frozen tumor samples	Allele-specific PCR (TaqMan), Sanger sequencing, or next-generation sequencing	Need for further clinical studies to provide additional evidence regarding the potential use of POLE/POLD1 mutations as molecular markers for treatment of CRC with ICIs
Tumor microenvironment markers	PD-L1 expression	Formalin-fixed, paraffin-embedded tumor samples	Immunohistochemistry	Critical need to establish a standardized set of criteria and a scoring system to accurately detect PD-L1 expression
	Immunoscore	Formalin-fixed, paraffin-embedded tumor samples	Immunohistochemistry	Need for standardized quality control measures and incorporation of additional cell subtypes, such as macrophages and neutrophils, with critical functions
Hematology-related markers	Plasma TMB	Blood samples for circulating cell-free DNA	Whole-exome sequencing, panel sequencing	Need for additional prospective trials to investigate and emphasize non-invasive markers enabling a reduced risk of diagnosis
	Inflammation-related markers	Peripheral blood	Blood cell counting with a hematological analyzer	Non-invasive markers including NLR, MLR, and PLR; need for multicenter prospective experimental validation and identification of the exact mechanisms underlying the relationship between these markers and treatment response
Gut microbiota	*Fusobacterium nucleatum* and others	Fecal samples	16S rDNA sequencing or metagenomic sequencing	Need to increase the sample size of individual cohorts, ensure prolonged follow-up, standardize protocols across diverse cohorts, and use more systematic and comprehensive analytical approaches

## Genomic markers

### Deficient mismatch repair/microsatellite instability

Deficient mismatch repair/microsatellite instability (dMMR/MSI) is one of the most important biomarker used to determine the efficacy of treatment of CRC with ICIs. Approximately 10%–15% of patients with sporadic CRC have dMMR/MSI^1^ The accumulation of DNA mutations in tumors in these patients can lead to neoantigen production and enhanced tumor immunogenicity, thus stimulating immune cell infiltration and anti-tumor immune responses^2^.

The KEYNOTE-016 clinical trial has assessed the relationship between MMR status and pembrolizumab treatment efficacy in metastatic tumors. In that study, individuals with dMMR/MSI CRC had an immune-related objective response rate (ORR) as high as 40%, whereas no efficacy was observed in individuals with proficient mismatch repair/microsatellite stability (pMMR/MSS)^3^. Several important subsequent clinical trials were conducted in patients with dMMR/MSI. A cohort study from the Checkmate-142 trial has confirmed that nivolumab achieves durable response and disease control in treated patients with dMMR/MSI^4^. Additional cohort studies from CheckMate-142 have confirmed the significant clinical efficacy of nivolumab in combination with ipilimumab in patients with CRC with dMMR/MSI receiving standard therapy^5,6^. Subsequently, KEYNOTE-164 confirmed the antitumor activity of pembrolizumab in previously treated dMMR/MSI CRC^7^. Furthermore, KEYNOTE-177 has indicated that pembrolizumab is an effective first-line therapy for dMMR/MSI patients with CRC^8,9^.

In 2017, U.S. Food & Drug Administration (FDA), on the basis of data from the KEYNOTE-016 and 164 studies, approved pembrolizumab for treating advanced and metastatic dMMR/MSI tumors. Moreover, in the same year, the FDA approved nivolumab as a second-line treatment for individuals with dMMR/MSI metastatic CRC. On the basis of data from the KEYNOTE-177 trial substantiating the administration of pembrolizumab as a first-line treatment, the FDA approved its use in treating dMMR/MSI CRC in 2020. These trials have underscored the importance of dMMR/MSI as a predictive marker for the treatment of CRC with ICIs.

### Tumor mutation burden (TMB)

TMB is the total number of somatic gene base substitutions, insertions, or deletions per million bases^10^. High TMB is associated with elevated neoantigen load and tumor-infiltrating lymphocytes. These factors contribute to heightened tumor immunogenicity, thus enhancing responsiveness to treatment with ICIs^11^.

The association between TMB and treatment outcomes has been explored in KEYNOTE-158, focusing on individuals with advanced solid tumors receiving pembrolizumab. The study used a threshold of 10 mutations per megabase (mut/Mb) and reported ORR values of 29% and 6% in the high and low TMB groups, respectively^12^. Friedman et al.^13^ have evaluated the association between TMB and response to treatment with atezolizumab (a PD-L1 inhibitor) in 90 patients with various tumor types (including colorectal cancer). The data indicated an ORR of 38.1% in 42 individuals with TMB ≥ 16 mut/Mb, in contrast to 2.1% in 48 individuals with TMB < 16 mut/Mb. In another study, Schrock et al.^14^ have highlighted the value of TMB in predicting ICI treatment response in dMMR/MSI CRC. Among individuals receiving ICIs, responders exhibited a median TMB of 54 mut/Mb, whereas non-responders had a median TMB of 29 mut/Mb. On the basis of a threshold of 37–41 mut/Mb distinguishing between high and low TMB patient subgroups, all 13 individuals in the high TMB group responded to treatment with ICIs. In contrast, 66.7% (6/9) of individuals in the low TMB group experienced disease progression. The REGONIVO trial has evaluated the efficacy of regorafenib in combination with nivolumab for advanced CRC. According to a cut-off value with the top quartile the ORRs were 50.0% and 35.3% in 25 patients with CRC with high and low TMB, respectively. Furthermore, the trial revealed longer median progression-free survival (PFS) in the high TMB group (12.5 months) than the low TMB group (7.9 months)^15^. These results have confirmed TMB’s potential as a valid biomarker for predicting the response of patients with tumors to ICI therapy.

The integration of TMB as a prognostic biomarker into standard clinical practice has encountered substantial obstacles. These challenges stem from discrepancies in TMB estimates obtained from the diverse DNA sequencing methods applied to identical tissue samples, as well as the absence of a consensus regarding the optimal concordance of TMB values across multiple platforms^16^. Notably, the thresholds used for determining patients with high and low TMB vary among studies. Thus a consensus must be reached regarding reasonable cut-off values to distinguish patients on the basis of TMB.

### DNA polymerase ε or polymerase δ1 mutation

The proofreading role of DNA polymerase ε (POLE) or polymerase δ1 (POLD1) is essential for maintaining DNA replication fidelity. POLE/POLD1 mutation-induced dysfunction of the DNA damage response system is a notable contributor to CRC development. Patients with CRC carrying mutations often show highly immunogenicity, with high levels of lymphocytic infiltration, cellular effector molecule expression, and favorable prognosis^17^. A cohort analysis in 47,721 individuals with various types of tumors^18^ has found a POLE/POLD1 mutation rate of 7.37% in CRC. The patients carrying these mutations had a significantly higher TMB than those without mutations. In the ICI treatment cohort, the individuals with POLE or POLD1 mutation had notably longer OS (34 months *vs*. 18 months). Furthermore, multifactorial analysis demonstrated that the POLE/POLD1 mutations serve as independent markers to identify patients benefiting from ICI treatment. Gong et al.^19^ have documented a case of a patient with MSS CRC carrying a POLE mutation, who exhibited a durable clinical response to pembrolizumab treatment. Data suggest that POLE mutations may be predictive of the response to ICIs in patients with MSS CRC. The consideration of POLE/POLD1 mutations as molecular markers for ICI treatment in CRC awaits further evidence from clinical studies.

## Tumor microenvironment markers

### Expression of PD-L1

The application of PD-L1 as a molecular marker for predicting the efficacy of ICI treatment has been demonstrated in various tumors^20^ but remains controversial in CRC. In the REGONIVO trial, the ORR for individuals with CRC with a PD-L1 combined positive score < 1 and ≥ 1 was 25% and 43.8%, respectively^15^. Although that study assessed a relatively small sample, the data indicate that individuals with high PD-L1 expression are likely to experience more favorable ICI treatment effects. However, contrasting results have been observed in the KEYNOTE-016 study, which has indicated no notable association between PD-L1 expression and the PFS or OS of individuals treated with pembrolizumab^3^. Checkmate-142 also has not revealed any notable association of PD-L1 expression on tumor cells with immunotherapeutic response^4^. The KEYNOTE-028 study enrolled 23 patients with CRC with positive PD-L1, wherein only one patient achieved a tumor response. Notably, the molecular typing of this particular individual was MSI^21^. Therefore, the predictive value of PD-L1 must be reconsidered.

The reasons for the variance in the results among trials is attributable to differences in the immunohistochemical assay processes and scoring systems. In addition, the predictive value of PD-L1 in ICI treatment is limited by the temporal and spatial heterogeneity of PD-L1 expression within tumors and vulnerability to disease progression and treatment modalities. Further research is essential to determine consistent criteria for detecting PD-L1 expression and to further assess and validate the effects of PD-L1 on ICI treatment efficacy and patient prognosis^20^.

### Tumor infiltrating immune cells

Tumor infiltrating lymphocytes (TILs) are an important predictor of prognosis in individuals with CRC^22^. The therapeutic effects of ICIs depend on the tumor-restricting effects of TILs. Higher levels of TILs are significantly correlated with treatment response and survival benefits in patients with CRC treated with ICIs^23^, thus suggesting that TILs may be a predictive indicator for assessing the efficacy of ICIs. Recent studies have proposed the Immunoscore as a tool for predicting recurrence and survival in patients with CRC. The Immunoscore quantifies various features, such as the densities and locations of different cell types in the immune microenvironment. The Immunoscore has been demonstrated to be superior to TNM staging and MSI status in predicting patient survival and recurrence^24,25^. Furthermore, research based on large sample data has demonstrated the ability of the Immunoscore to predict adjuvant chemotherapy efficacy in colon cancer^26^.

As a novel tumor staging scheme, the Immunoscore provides an ideal solution for improving patient stratification, predicting tumor patient regression, and assessing treatment outcomes. However, the lack of well-defined cell density threshold values hinders direct integration and comparison of results across different studies, thereby restricting broader applications of the Immunoscore in prospective clinical studies. Additionally, a need exists for standardized quality control measures and the inclusion of more cell subtypes with important functions, such as macrophages and neutrophils. The Immunoscore method and its applications are anticipated to undergo further refinement and optimization as clinical studies continue to progress and accumulate, thereby paving the way to widespread clinical application.

## Hematology-related markers

The use of reliable non-invasive markers is essential for facilitating easier, risk-lower diagnosis. Studies have explored blood TMB to predict the treatment efficacy of ICIs. The CCTG CO.26 trial has examined plasma TMB on the basis of circulating cell-free DNA in blood samples, reported as variations per megabase (vts/Mb), by using the GuardantOMNI algorithm in individuals treated with tremelimumab in combination with durvalumab. The resulting data have indicated that individuals with elevated TMB (≥ 28 vts/Mb) experienced a notable OS benefit, thus suggesting that plasma TMB is a valid marker for screening potential beneficiaries of ICIs^27^.

Recent studies have also focused on the value of inflammation-related markers in predicting the therapeutic efficacy of tumor ICIs^28^. For instance, Fan et al.^29^ have found significantly higher disease control rates in individuals receiving anti-PD-1 therapy with a neutrophil-to-lymphocyte ratio (NLR) < 5 than an NLR > 5. In addition, a monocyte-to-lymphocyte ratio (MLR) < 0.31 has been associated with longer PFS and OS. A platelet-to-lymphocyte ratio (PLR) < 135 has been associated with greater occurrence of immune-related adverse events. These results have indicated the potential clinical utility of NLR, MLR, and PLR in predicting survival or the risk of immune-related adverse events in individuals with advanced CRC. Nonetheless, further prospective trials are needed to explore and highlight new and improved biomarkers. In addition, the precise mechanism underlying the relationship between immune markers and treatment response must be clarified.

## Gut microbiome

The enduring coevolution of the host and gut microbiota has engendered a mutual reliance. Increasing evidence indicates that disruptions in the gut microbiota affect the immunotherapeutic responses of diverse tumors via interactions with the host immune system^30^. Increasing attention is being directed toward potential use of the gut microbiota as a predictive biomarker and enhancer of ICI treatment efficacy^31^. One study recruiting 74 individuals diagnosed with advanced gastrointestinal tumors treated with ICIs has demonstrated an elevated *Prevotella/Bacteroides* ratio among patients exhibiting favorable responses. Furthermore, a specific subgroup of responders displayed a notably elevated prevalence of *Prevotella*, *Ruminococcaceae*, and *Lachnospiraceae*^32^. The role of *Fusobacterium nucleatum* as the primary pathogenic bacterium in CRC has been substantiated. Recently, patients with advanced CRC who exhibited unresponsiveness to immunotherapy have been reported to show an elevated fecal abundance of *Fusobacterium nucleatum*, which correlates with an unfavorable prognosis^33^.

Although the gut microbiota is emerging as a major determinant influencing the efficacy of tumor immunotherapy, additional investigations including extensive cohort studies and clinical trials are imperative. As flora sequencing technology advances, the gut microbiota is anticipated to serve as a prognostic biomarker for CRC immunotherapy. Before treatment, fecal microbiota sequencing has the potential to predict treatment outcomes and aid in treatment decision-making and planning, through quantifying community abundance and the relative proportions of bacteria deemed beneficial or harmful.

## Discussion

Predictive biomarkers aid in not only identifying the individuals who stand to benefit most from immunotherapy, but also avoiding unnecessary costs, accelerated progression, and possible severe toxicity resulting from treatment of non-responders. Currently, biomarkers remain a major challenge in immunotherapy. Tumor tissue accessibility, spatial and temporal heterogeneity, and inconsistent evaluation criteria remain major obstacles that must be addressed through the development of more standardized evaluation and detection tools in the future.

Importantly, no single predictive biomarker can effectively identify the beneficiary population, and each biomarker may be limited in certain aspects. The use of combined assays or the development of validated predictive models may enhance predictive sensitivity. Recent studies have introduced a theoretical framework known as the cancer immunogram^34^, which amalgamates various factors into a composite biomarker encompassing variables such as TMB, PD-L1 expression, specific genes associated with immune response or resistance, immune infiltration, and the microbiome. Further investigation is warranted to explore the potential of this integrated model in enhancing the diagnosis and treatment efficiency of CRC. However, efforts must be made to avoid excessively limiting the pool of eligible patients for immunotherapy.

In addition, additional highly sensitive and specific therapeutic markers for ICIs must be identified, for example, through the combined application of high-precision genomic tools such as transcriptomics, proteomics and microbiomics, thereby further enhancing the efficacy of ICIs and improving patient prognosis.

## References

[1] Richman S (2015). Deficient mismatch repair: read all about it (Review). Int J Oncol.

[2] Maby P, Tougeron D, Hamieh M, Mlecnik B, Kora H, Bindea G (2015). Correlation between density of CD8+ T-cell infiltrate in microsatellite unstable colorectal cancers and frameshift mutations: a rationale for personalized immunotherapy. Cancer Res.

[3] Le DT, Uram JN, Wang H, Bartlett BR, Kemberling H, Eyring AD (2015). PD-1 blockade in tumors with mismatch-repair deficiency. N Engl J Med.

[4] Overman MJ, McDermott R, Leach JL, Lonardi S, Lenz HJ, Morse MA (2017). Nivolumab in patients with metastatic DNA mismatch repair-deficient or microsatellite instability-high colorectal cancer (CheckMate 142): an open-label, multicentre, phase 2 study. Lancet Oncol.

[5] Overman MJ, Lonardi S, Wong KYM, Lenz HJ, Gelsomino F, Aglietta M (2018). Durable clinical benefit with nivolumab plus ipilimumab in DNA mismatch repair-deficient/microsatellite instability-high metastatic colorectal cancer. J Clin Oncol.

[6] André T, Lonardi S, Wong KYM, Lenz HJ, Gelsomino F, Aglietta M (2022). Nivolumab plus low-dose ipilimumab in previously treated patients with microsatellite instability-high/mismatch repair-deficient metastatic colorectal cancer: 4-year follow-up from CheckMate 142. Ann Oncol.

[7] Le DT, Kim TW, Van Cutsem E, Geva R, Jäger D, Hara H (2020). Phase II open-label study of pembrolizumab in treatment-refractory, microsatellite instability-high/mismatch repair-deficient metastatic colorectal cancer: KEYNOTE-164. J Clin Oncol.

[8] Diaz LA, Shiu KK, Kim TW, Jensen BV, Jensen LH, Punt C (2022). Pembrolizumab versus chemotherapy for microsatellite instability-high or mismatch repair-deficient metastatic colorectal cancer (KEYNOTE-177): final analysis of a randomised, open-label, phase 3 study. Lancet Oncol.

[9] André T, Shiu KK, Kim TW, Jensen BV, Jensen LH, Punt C (2020). Pembrolizumab in microsatellite-instability-high advanced colorectal cancer. N Engl J Med.

[10] Fancello L, Gandini S, Pelicci PG, Mazzarella L (2019). Tumor mutational burden quantification from targeted gene panels: major advancements and challenges. J Immunother Cancer.

[11] McGranahan N, Furness AJ, Rosenthal R, Ramskov S, Lyngaa R, Saini SK (2016). Clonal neoantigens elicit T cell immunoreactivity and sensitivity to immune checkpoint blockade. Science.

[12] Marabelle A, Fakih M, Lopez J, Shah M, Shapira-Frommer R, Nakagawa K (2020). Association of tumour mutational burden with outcomes in patients with advanced solid tumours treated with pembrolizumab: prospective biomarker analysis of the multicohort, open-label, phase 2 KEYNOTE-158 study. Lancet Oncol.

[13] Friedman CF, Hainsworth JD, Kurzrock R, Spigel DR, Burris HA, Sweeney CJ (2022). Atezolizumab treatment of tumors with high tumor mutational burden from MyPathway, a multicenter, open-label, phase IIa multiple basket study. Cancer Discov.

[14] Schrock AB, Ouyang C, Sandhu J, Sokol E, Jin D, Ross JS (2019). Tumor mutational burden is predictive of response to immune checkpoint inhibitors in MSI-high metastatic colorectal cancer. Ann Oncol.

[15] Fukuoka S, Hara H, Takahashi N, Kojima T, Kawazoe A, Asayama M (2020). Regorafenib plus nivolumab in patients with advanced gastric or colorectal cancer: an open-label, dose-escalation, and dose-expansion phase Ib trial (REGONIVO, EPOC1603). J Clin Oncol.

[16] Ricciuti B, Awad MM (2023). Atezolizumab plus bevacizumab in TMB-high non-small cell lung cancers-the hunt for predictive biomarkers to optimize treatment selection. JAMA Oncol.

[17] Domingo E, Freeman-Mills L, Rayner E, Glaire M, Briggs S, Vermeulen L (2016). Somatic POLE proofreading domain mutation, immune response, and prognosis in colorectal cancer: a retrospective, pooled biomarker study. Lancet Gastroenterol Hepatol.

[18] Wang F, Zhao Q, Wang YN, Jin Y, He MM, Liu ZX (2019). Evaluation of POLE and POLD1 mutations as biomarkers for immunotherapy outcomes across multiple cancer types. JAMA Oncol.

[19] Gong J, Wang C, Lee PP, Chu P, Fakih M (2017). Response to PD-1 blockade in microsatellite stable metastatic colorectal cancer harboring a POLE mutation. J Natl Compr Canc Netw.

[20] Doroshow DB, Bhalla S, Beasley MB, Sholl LM, Kerr KM, Gnjatic S (2021). PD-L1 as a biomarker of response to immune-checkpoint inhibitors. Nat Rev Clin Oncol.

[21] O’Neil BH, Wallmark JM, Lorente D, Elez E, Raimbourg J, Gomez-Roca C (2017). Safety and antitumor activity of the anti-PD-1 antibody pembrolizumab in patients with advanced colorectal carcinoma. PLoS One.

[22] Rozek LS, Schmit SL, Greenson JK, Tomsho LP, Rennert HS, Rennert G (2016). Tumor-infiltrating lymphocytes, Crohn’s-like lymphoid reaction, and survival from colorectal cancer. J Natl Cancer Inst.

[23] Loupakis F, Depetris I, Biason P, Intini R, Prete AA, Leone F (2020). Prediction of benefit from checkpoint inhibitors in mismatch repair deficient metastatic colorectal cancer: role of tumor infiltrating lymphocytes. Oncologist.

[24] Pagès F, Mlecnik B, Marliot F, Bindea G, Ou FS, Bifulco C (2018). International validation of the consensus immunoscore for the classification of colon cancer: a prognostic and accuracy study. Lancet.

[25] Mlecnik B, Bindea G, Angell HK, Maby P, Angelova M, Tougeron D (2016). Integrative analyses of colorectal cancer show immunoscore is a stronger predictor of patient survival than microsatellite instability. Immunity.

[26] Pagès F, André T, Taieb J, Vernerey D, Henriques J, Borg C (2020). Prognostic and predictive value of the Immunoscore in stage III colon cancer patients treated with oxaliplatin in the prospective IDEA France PRODIGE-GERCOR cohort study. Ann Oncol.

[27] Chen EX, Jonker DJ, Loree JM, Kennecke HF, Berry SR, Couture F (2020). Effect of combined immune checkpoint inhibition vs best supportive care alone in patients with advanced colorectal cancer: the Canadian Cancer Trials Group CO.26 study. JAMA Oncol.

[28] Sui Q, Zhang X, Chen C, Tang J, Yu J, Li W (2022). Inflammation promotes resistance to immune checkpoint inhibitors in high microsatellite instability colorectal cancer. Nat Commun.

[29] Fan X, Wang D, Zhang W, Liu J, Liu C, Li Q (2021). Inflammatory markers predict survival in patients with advanced gastric and colorectal cancers receiving anti-PD-1 therapy. Front Cell Dev Biol.

[30] Routy B, Le Chatelier E, Derosa L, Duong CPM, Alou MT, Daillère R (2018). Gut microbiome influences efficacy of PD-1-based immunotherapy against epithelial tumors. Science.

[31] Si H, Yang Q, Hu H, Ding C, Wang H, Lin X (2021). Colorectal cancer occurrence and treatment based on changes in intestinal flora. Semin Cancer Biol.

[32] Peng Z, Cheng S, Kou Y, Wang Z, Jin R, Hu H (2020). The gut microbiome is associated with clinical response to anti-PD-1/PD-L1 immunotherapy in gastrointestinal cancer. Cancer Immunol Res.

[33] Jiang SS, Xie YL, Xiao XY, Kang ZR, Lin XL, Zhang L (2023). Fusobacterium nucleatum-derived succinic acid induces tumor resistance to immunotherapy in colorectal cancer. Cell Host Microbe.

[34] Blank CU, Haanen JB, Ribas A, Schumacher TN (2016). Cancer immunology. The “cancer immunogram”. Science.

